# Data for analysis of mannose-6-phosphate glycans labeled with fluorescent tags

**DOI:** 10.1016/j.dib.2016.04.042

**Published:** 2016-04-23

**Authors:** Ji-Yeon Kang, Ohsuk Kwon, Jin Young Gil, Doo-Byoung Oh

**Affiliations:** a Synthetic Biology and Bioengineering Research Center, Korea Research Institute of Bioscience and Biotechnology (KRIBB), Daejeon, Republic of Korea; b Biosystems and Bioengineering Program, University of Science and Technology (UST), Daejeon, Republic of Korea

## Abstract

Mannose-6-phosphate (M-6-P) glycan plays an important role in lysosomal targeting of most therapeutic enzymes for treatment of lysosomal storage diseases. This article provides data for the analysis of M-6-P glycans by high-performance liquid chromatography (HPLC) and matrix-assisted laser desorption/ionization time-of-flight (MALDI-TOF) mass spectrometry. The identities of M-6-P glycan peaks in HPLC profile were confirmed by measuring the masses of the collected peak eluates. The performances of three fluorescent tags (2-aminobenzoic acid [2-AA], 2-aminobenzamide [2-AB], and 3-(acetyl-amino)-6-aminoacridine [AA-Ac]) were compared focusing on the analysis of bi-phosphorylated glycan (containing two M-6-Ps). The bi-phosphorylated glycan analysis is highly affected by the attached fluorescent tag and the hydrophilicity of elution solvent used in HPLC. The data in this article is associated with the research article published in “*Comparison of fluorescent tags for analysis of mannose-6-phosphate glycans*” (Kang et al., 2016 [1]).

**Specifications Table**

**Table:** 

Subject area	*Biology, Chemistry*
More specific subject area	*Glycobiology, Analytical biochemistry*
Type of data	*Image, Figure (HPLC profile and Mass spectra), Graph*
How data was acquired	*M-6-P glycans labeled with fluorescent tags were analyzed by HPLC and MALDI-TOF mass spectrometry*
Data format	*Analyzed*
Experimental factors	*Mannosylphosphoryalted N-glycans obtained from the glyco-engineered yeast were converted to M-6-P glycans by the uncapping process using mild acid hydrolysis*
Experimental features	*M-6-P glycan peaks in HPLC analysis were identified by measuring the masses of the collected peak eluates.*
Data source location	*Daejeon, Republic of Korea*
Data accessibility	*The data are supplied with this article*

## Value of the data

• The M-6-P glycan analysis data, which were obtained by HPLC and mass spectrometry after the labeling of three commonly used fluorescent tags (2-AA, 2-AB and AA-Ac), can be used for the comparison of their performances.
• The hydrophilicity-optimized elution solvent in HPLC analysis can be used for proper detection and quantification of the bi-phosphorylated glycan (containing two M-6-Ps).
• Careful analysis and interpretation are required when analyzing mannosylphosphorylated glycans by using the MALDI-TOF mass spectrometry because the acidic matrix preparation condition can convert some of them to M-6-P glycans.

## 1 Data

After the optimization of HPLC condition for the analysis of bi-phosphoyrlated glycan (containing two M-6-Ps), the 2-aminobenzoic acid (2-AA)-labeled glycan peaks were identified by measuring the masses of the collected peak eluates using matrix-assisted laser desorption/ionization time-of-flight (MALDI-TOF) mass spectrometry (Fig. 2). In contrast to 2-AA labeling, 2-aminobenzamide (2-AB) and 3-(acetyl-amino)-6-aminoacridine (AA-Ac) labelings enabled the detection of bi-phosphorylated glycan in HPLC without hydrophilicity optimization of elution solvent (Fig. 3). Fig. 4 shows the data of MALDI-TOF mass spectrometry analysis for the conversions of mannosylphosphorylated glycans to M-6-P glycans after the labeling of three fluorescent tags in order to compare their performances increasing detection sensitivity.

## 2 Experimental design, materials, and methods

### 2.1 *N*-glycan preparation and fluorescent tag labeling

Most of the therapeutic enzymes for treatment of lysosomal storage diseases require M-6-P glycan, which are recognized by the M-6-P receptor on the plasma membrane for lysosomal targeting [2]. Although yeasts do not have M-6-P glycans in nature, some of their glycans containing mannosylphosphate residue can be converted to M-6-P glycans through the uncapping process to remove the outer mannose residue. Recently, we developed a glyco-engineered *Saccharomyces cerevisiae och1Δmnn1Δ/YlMpo1* strain generating high content of mannosylphosphorylated glycans which can be converted to M-6-P glycans [3]. From this yeast, *N*-glycans were prepared as described previously [3]. Briefly, yeast cell wall mannoproteins were extracted by using hot citrate buffer and subsequent precipitation with ethanol. The obtained mannoproteins were digested to glycopeptides, followed by glycopeptide purification using a C18 Sep-Pak cartridge as previously described [4]. *N*-glycans were released from the purified glycopeptide by Peptide-*N*-glycosidase F (New England Biolabs, Ipswich, MA, USA) treatment at 37 °C overnight and purified by solid-phase extraction using graphitized carbon (Alltech, Lexington, MA, USA) [5].

**Fig. 1:**
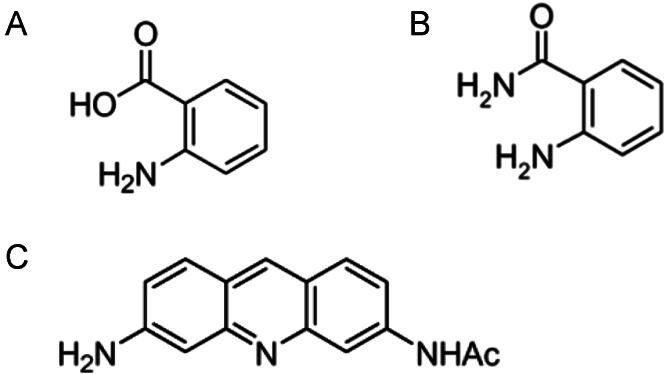
Chemical structure of 2-AA, 2-AB, and AA-Ac tags. Structures of 2-AA (A), 2-AB (B), and AA-Ac (C) are represented.

The purified glycans were fluorescently labeled with 2-AA, 2-AB, or AA-Ac; the structures of these fluorescent tags are represented in Fig. 1. 2-AA and 2-AB were purchased from Sigma-Aldrich (St. Louis, MO, USA), while AA-Ac was obtained from Ludger Ltd. (Oxfordshire, UK). The labeling reagent was freshly prepared by dissolving 6 mg of 2-AA, 2-AB, or AA-Ac in 100 μl of dimethyl sulfoxide/acetic acid (7:3, v/v) containing 1 M of sodium cyanoborohydride. Dried glycans were dissolved in 5 μl of each labeling reagent and mixed thoroughly. The reaction mixture for 2-AA or 2-AB labeling was incubated at 37 °C overnight, while the one for AA-Ac labeling was incubated at 80 °C for 30 min. The resulting 2-AA-, 2-AB-, or AA-Ac labeled glycans were purified from unreacted labeling reagents using cyano-SPE cartridge (Agilent Technologies, Santa Clara, CA) as described previously [5].

### 2.2 Identification of 2-AA-labeled glycan peaks in HPLC analysis

**Fig. 2:**
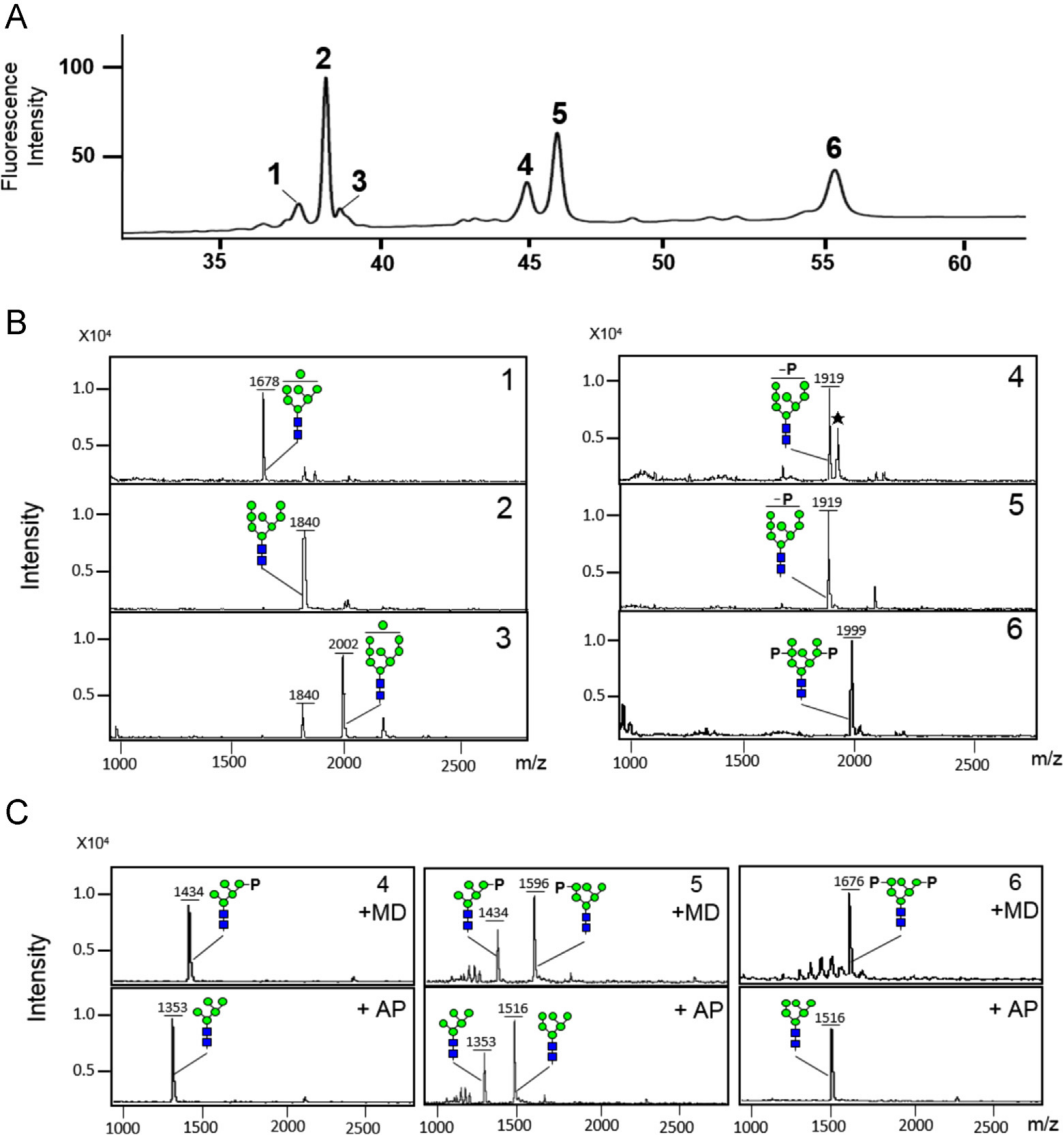
The peaks of M-6-P glycans labeled with 2-AA were identified from the mass spectra of collected peak eluates. (A) All of the 2-AA-labeled glycan peaks (1–6) in the HPLC profile were collected. (B) Mass of each collected peak (peak number is represented in each mass spectrum) was analyzed by using MALDI-TOF mass spectrometry. (C) After α(1,2)-mannosidase digestion (+MD) and the subsequent alkaline phosphatase digestion (+AP), the masses of glycan peaks 4, 5, and 6 were analyzed. Symbols used for glycans are those suggested by the Consortium for Functional Glycomics (http://www.functionalglycomics.org/). Green circle: mannose, blue square: GlcNAc, P: phosphate.

Separation of 2-AA-labeled glycans was performed with a Shodex Asahipak NH2P-50 amine column (5 μm, 4.6 mm×250 mm) purchased from Showa Denko (Tokyo, Japan) using a Waters Alliance system equipped with a Waters 2475 fluorescence detector (Milford, MA, USA). Solvent A consisted of acetonitrile containing 2% acetic acid and 1% tetrahydrofuran. Solvent B consisted of 5% acetic acid, 3% triethylamine, and 1% tetrahydrofuran in water, while solvent B-h consisted of 10% acetic acid, 6% triethylamine, and 1% tetrahydrofuran. The column was equilibrated with 90% A and 10% B (or B-h). After injecting a 10 µl sample, elution was carried out with a linear gradient from 90% A and 10% B to 10% A and 90% B at a flow rate of 1 ml/min for 70 min at 50 °C. Fluorescence was monitored with excitation (Ex) and emission (Em) wavelengths, 360 and 425 nm, respectively. The eluted fractions were collected and their masses were identified by MALDI-TOF mass spectrometry (Fig. 2). On the amine column HPLC, the AA-labeled glycans were separated to neutral mannose-type (Man_7–9_GlcNAc_2_; peaks 1–3), mono-phosphorylated (P-Man_8_GlcNAc_2_; peaks 4 and 5), and bi-phosphorylated (P_2_-Man_8_GlcNAc_2_; peak 6) in accordance with their charge and size. Their identities were further confirmed by the successive digestions of α(1,2)-mannosidase (Prozyme, Haywoard, CA) and calf intestinal alkaline phosphatase (Takara, Tokyo, Japan) as previously described [3]. The α(1,2)-mannosidase treatment converted high-mannose type glycans Man_7–9_GlcNAc_2_ (*m*/*z* 1678, 1840, and 2002) to Man_5_GlcNAc_2_ (*m*/*z* 1353) (data not shown). Although the peaks 4 and 5 (mono-phosphorylated, P-Man_8_GlcNAc_2_) had the same molecular weight 1919, they were converted to two molecular weights 1434 (P-Man_5_GlcNAc_2_) and 1596 (P-Man_6_GlcNAc_2_) after the α(1,2)-mannosidase treatment (4+MD and 5+MD in Fig. 2C). The peak 4 glycan appeared to have phosphate group at the α(1,6)-branch of Man_8_GlcNAc_2_ whereas the major phosphorylation site of peak 5 glycan was at the α(1,3)-branch; α(1,2)-mannosidase did not remove the terminal α(1,2)-mannose residue at the α(1,3)-branch due to the existence of attached phosphate group, as previously described [6], [7]. The existences of one phosphate group in peak 4 and 5 glycans were confirmed by alkaline phosphatase digestion (4+MD+AP and 5+MD+AP in Fig. 2C). The bi-phosphorylated glycan (peak 6, *m*/*z* 1999) was converted to P_2_-Man_6_GlcNAc_2_ (*m*/*z* 1676) by α(1,2)-mannosidase digestion (6+MD in Fig. 2C) and further to Man_6_GlcNAc_2_ (*m*/*z* 1516) by the subsequent alkaline phosphatase digestion (6+MD+AP in Fig. 2C), indicating the existences of two phosphate groups.

### 2.3 HPLC analysis of 2-AB- and AA-Ac-labeled glycans

**Fig. 3:**
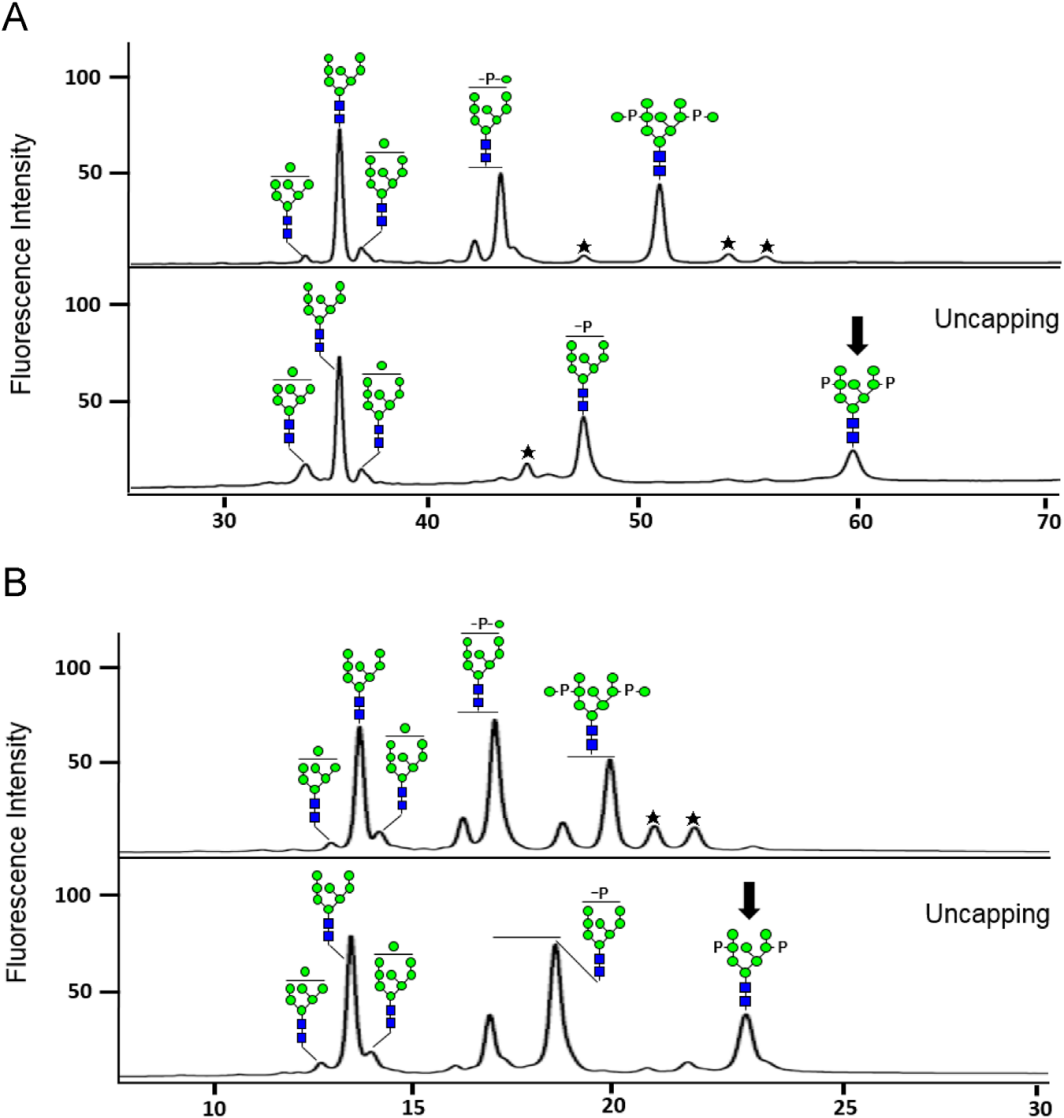
Detection of bi-phosphorylated *N*-glycans labeled with 2-AB and AA-Ac. M-6-P glycans generated from mannosylphosphorylated glycans by uncapping MAH were analyzed after 2-AB (A) and AA-Ac (B) labeling. Symbols are identical to those used in Fig. 2.

M-6-P glycans fluorescently labeled with 2-AB or AA-Ac were analyzed by HPLC using the same conditions described in Section 2.2. Wavelengths for fluorescence detection were adjusted for 2-AB (Ex 330 and Em 420 nm) and AA-Ac (Ex 442 and Em 525 nm). Fig. 3 shows the profiles of 2-AB- and AA-Ac-labeled glycans obtained from HPLC analysis using solvents A and B (without the increased hydrophilicity). Notably, the bi-phosphorylated glycan was detected at 58 and 23 min in 2-AB- and AA-Ac-labeled glycan profiles in this condition, which is in sharp contrast with the result for 2-AA-labeled glycans requiring the use of elution solvent B-h (with the increased hydrophilicity) for the detection of bi-phosphorylated glycan (see the Fig. S1A and B in Ref. [1]). All 2-AB- and AA-Ac-labeled M-6-P glycans eluted later in the HPLC analysis using solvents A and B compared with the analysis using solvents A and B-h (see the Fig. S1C in Ref. [1]); especially, the elution times of bi-phosphorylated glycans labeled with 2-AB and AA-Ac on using solvent B were 8 and 2 min later than those on using solvent B-h (52 and 21 min).

### 2.4 MALDI-TOF mass spectrometry analysis of M-6-P glycan

**Fig. 4:**
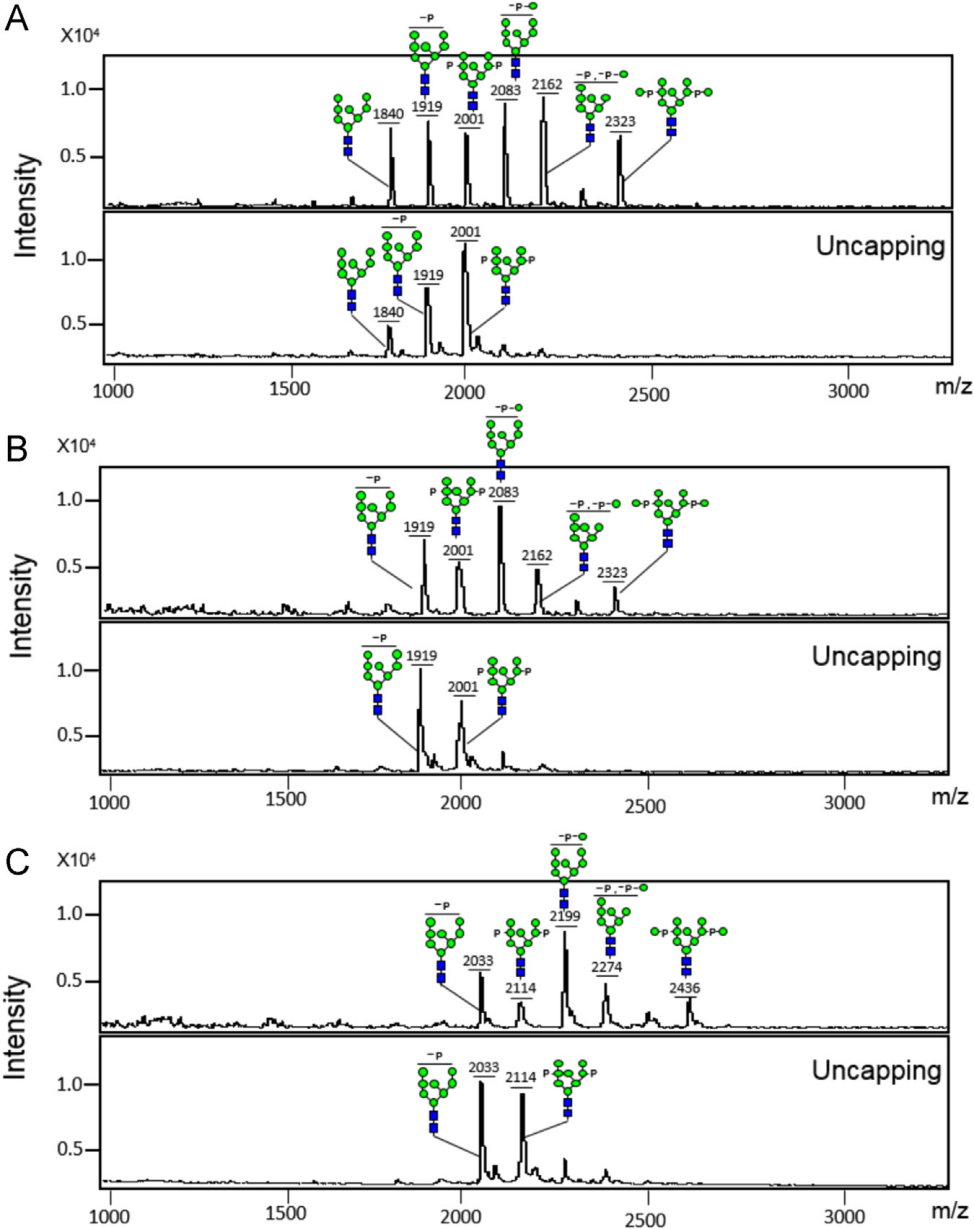
Conversions of mannosylphosphorylated glycans to M-6-P glycans were analyzed by MALDI-TOF mass spectrometry. After 2-AA (A), 2-AB (B), and AA-Ac (C) labeling, the masses of mannosylphsphorylated *N*-glycans (upper panels) and M-6-P glycans generated through uncapping (lower panels) were analyzed. Notably, several glycans containing M-6-P were observed in the mass spectra of mannosylphsphorylated *N*-glycans. It seems that the matrix preparation condition for mass analysis, which is acidic, induces partial uncapping of mannosylphosphorylated *N*-glycans. Symbols are identical to those used in Fig. 2.

Glycans were analyzed using a Microflex MALDI-TOF mass spectrometry (Bruker Daltonik, GmbH, Bremen, Germany) as previously described [5] with a slight modification. Briefly, the labeled glycans were spotted on the MALDI MSP96 polished steel chip (Bruker Daltonik) and then 6-Aza-2-thiothymine (ATT)/2,5-dihydroxybenzoic acid (DHB) matrix solution was added, followed by drying in air. All mass spectra were acquired in a linear negative ion mode using the method recommended by the manufacturer because the mannosylphosphorylated and phosphorylated glycans have negative charges in their phosphate groups. Due to the low resolution of linear negative mode, we experienced some deviations (up to ~2 Da) from theoretical mass values. Fig. 4 shows the analysis results of 2-AA-, 2-AB-, or AA-Ac-labeled mannosylphosphorylated glycans and their uncapped forms containing M-6-Ps, which suggested that some of the mannosylphosphorylated glycans were uncapped during the acidic matrix preparation step.

## References

[1] Kang J.Y., Kwon O., Gil J.Y., Oh D.B. (2016). Comparison of fluorescent tags for analysis of mannose-6-phosphate glycans. Anal. Biochem..

[2] Oh D.B. (2015). Glyco-engineering strategies for the development of therapeutic enzymes with improved efficacy for the treatment of lysosomal storage diseases. BMB Rep..

[3] Gil J.Y., Park J.N., Lee K.J., Kang J.Y., Kim Y.H., Kim S., Kim S.Y., Kwon O., Lim Y.T., Kang H.A., Oh D.B. (2015). Increased mannosylphosphorylation of N-glycans by heterologous expression of YlMPO1 in glyco-engineered Saccharomyces cerevisiae for mannose-6-phosphate modification. J. Biotechnol..

[4] Lee K.J., Gil J.Y., Kim S.Y., Kwon O., Ko K., Kim D.I., Kim D.K., Kim H.H., Oh D.B. (2015). Molecular characterization of acidic peptide: N-glycanase from the dimorphic yeast Yarrowia lipolytica. J. Biochem..

[5] Mun J.Y., Lee K.J., Seo H., Sung M.S., Cho Y.S., Lee S.G., Kwon O., Oh D.B. (2013). Efficient adhesion-based plasma membrane isolation for cell surface N-glycan analysis. Anal. Chem..

[6] Wang X.H., Nakayama K.I., Shimma Y.I., Tanaka A., Jigami Y. (1997). MNN6, a member of the KRE2/MNT1 family, is the gene for mannosylphosphate transfer in *Saccharomyces cerevisiae*. J. Chem..

[7] Tiels P., Baranova E., Piens K., Visscher C.D., Pynaert G., Nerinckx W., Stout J., Fudalej F., Hulpiau P., Tännler S., Geysens S., Hecke A.V., Valevska A., Vervecken W., Remaut H., Callewaert N. (2012). A bacterial glycosidase enables mannose-6-phosphate modification and improved cellular uptake of yeast-produced recombinant human lysosomal enzymes. Nat. Biotechnol..

